# Antimicrobial susceptibility and minimum inhibitory concentration distribution of common clinically relevant non-tuberculous mycobacterial isolates from the respiratory tract

**DOI:** 10.1080/07853890.2022.2121984

**Published:** 2022-09-18

**Authors:** Guiqing He, Lianpeng Wu, Qingyong Zheng, Xiangao Jiang

**Affiliations:** a Department of Infectious Diseases, Wenzhou Central Hospital, The Dingli Clinical College of Wenzhou Medical University, The Second Affiliated Hospital of Shanghai University, Wenzhou, People’s Republic of China; b Laboratory of Infectious Diseases, Wenzhou Central Hospital, The Dingli Clinical College of Wenzhou Medical University, The Second Affiliated Hospital of Shanghai University, Wenzhou, People’s Republic of China; c Department of Clinical Laboratory, Wenzhou Central Hospital, The Dingli Clinical College of Wenzhou Medical University, The Second Affiliated Hospital of Shanghai University, Wenzhou, People‘s Repulic of China

**Keywords:** Non-tuberculous mycobacteria (NTM), *Mycobacterium avium* complex (MAC), *Mycobacterium abscessus* complex (MAB), *Mycobacterium kansasii* (*M. kansasii*), Drug susceptibility

## Abstract

**Objective:** To determine the minimum inhibitory concentration (MIC) distribution of antibacterial drugs and the susceptibility of non-tuberculous mycobacterial (NTM) isolates to provide a reference basis for the clinical selection of an effective starting regimen.

**Methods:** The common clinical isolates of NTM in the respiratory tract, which met the standards of the American Thoracic Society for NTM lung disease, were collected. The MICs of 81 isolates were determined using the microbroth dilution method (Thermo Fisher Scientific, USA), as recommended by the Clinical and Laboratory Standards Institute, USA.

**Results:** Included were 43 *Mycobacterium avium* complex (MAC) strains, 24 *M. abscessus* complex (MAB) strains, and 14 *M. kansasii* strains. The sensitivity rates of MAC to clarithromycin and amikacin were 81.4% and 79.1%, respectively, while the sensitivity rates to linezolid and moxifloxacin were only 20.9% and 9.3%; the MIC of rifabutin was the lowest (MIC50% was just 2 μg/mL). After incubation for 3–5 days, the sensitivity rate of MAB to clarithromycin was 87.5%; this decreased to 50% after 14 days’ incubation. Most of them were susceptible to amikacin (91.6%), and most were resistant to moxifloxacin (95.8%), ciprofloxacin (95.8%), imipenem (95.8%), amoxicillin/clavulanate (95.8%), tobramycin (79.1%), doxycycline (95.8%) and trimethoprim/sulfamethoxazole (95.8%). intermediate (83.3%) and resistant (16.7%) to cefoxitin. The susceptibility to linezolid was only 33.3%. The sensitivity and resistance breakpoints of tigecycline were set to ≤0.5 and ≥8 μg/mL, respectively, and the sensitivity and resistance rates were 50% and 0%, respectively. *M. kansasii* was susceptible to clarithromycin, amikacin, linezolid, moxifloxacin, rifampicin and rifabutin (100%).

**Discussion:** In Wenzhou, clarithromycin, amikacin and rifabutin have good antibacterial activity against MAC, while linezolid and moxifloxacin have high resistance. Amikacin and tigecycline have strong antibacterial activity against MAB, while most other antibacterial drugs are resistant to varying degrees. Most antibacterial drugs are susceptible to *M*. *kansasii* and have good antibacterial activity.

**Conclusion:** The identification of NTM species and the detection of their MICs have certain guiding values for the treatment of NTM lung disease.

**Key Message**
- The three most common respiratory non-tuberculous mycobacterial (NTM) isolates with clinical significance in the Wenzhou area were tested for drug susceptibility. The broth microdilution method was used to determine the minimum inhibitory concentration distribution of antibacterial drugs and the susceptibility of NTM isolates to provide a reference basis for the clinical selection of an effective starting regimen.

## 1. Introduction

In recent years, infections of non-tuberculous mycobacteria (NTM) have become increasingly prevalent and have emerged as an important public health problem. To date, the number of NTM species exceeds 190 [1]. Non-tuberculous mycobacteria can infect not only immunodeficient individuals but also immunocompetent individuals. Among the infected population, most NTM can cause pulmonary infections, and a few can cause extrapulmonary infections, which are a serious health hazard.

A recent prospective study in China [2] found that the isolation rate of respiratory NTM was 7.8% (530/6,766), of which 86.4% (458/530) met the diagnostic criteria for NTM lung disease, and only 13.6% (72/530) were considered colonisation. It has been reported that the 5-, 10- and 15-year mortality rates of NTM lung disease are 12.4%, 24.0% and 36.4%, respectively [3]. Therefore, NTM lung disease should not be neglected in clinical diagnosis and treatment.

A national study [2] showed that the top three NTM species in patients with NTM lung disease were *Mycobacterium avium* complex (MAC) (61.1%), *M. abscessus* complex (MAB) (23.1%) and *M. kansasii* (8.1%), accounting for 92.3% of cases of NTM lung disease. It is difficult to treat NTM lung disease, especially the MAC and MAB types, mainly because of the long treatment course, adverse effects, the combination of at least three or more drugs, low cure rates and high rates of recurrence and reinfection [1]. The most critical aspect of effective treatment is the development of an effective starting regimen, which varies with different NTM species and drug sensitivities. Apart from the known relatively clear correlation between antimicrobial susceptibility and treatment outcomes in NTM lung disease with amikacin and macrolides (clarithromycin and azithromycin) and the known correlation between the *in vitro* susceptibility of rifampicin and treatment outcomes in *M. kansasii*, the correlation between the minimum inhibitory concentration (MIC) of most other antimicrobial drugs and their *in vivo* efficacy remains unclear; however, the relationship can serve as a reference for starting combination regimens and guide the clinical selection of drugs [1].

The Clinical and Laboratory Standards Institute (CLSI) recommends broth microdilution susceptibility testing for NTM [4], with baseline and relapse/failure susceptibility testing for patients with clinically significant NTM isolates. In the present study, the three most common respiratory NTM isolates with clinical significance in the Wenzhou area were tested for drug sensitivity (*via* the broth microdilution method) to understand the MIC distribution of antibacterial drugs, the susceptibility of NTM isolates and to provide a reference basis for the clinical selection of an effective starting regimen.

## 2. Research subjects and methods

### 2.1. Study subjects

A total of 81 of the most common respiratory NTM isolates identified in the tuberculosis (TB) laboratory of our hospital, excluding duplicate strains (i.e. multiple strains from the same patient), were collected randomly between November 2019 and December 2021. All strains were obtained from our TB laboratory and from designated TB laboratories in all districts and counties of Wenzhou. Then, they were sent to the municipal central TB laboratory (i.e. our TB laboratory) for further strain identification. All strains selected for drug sensitivity testing met the American Thoracic Society’s (ATS) NTM lung disease diagnostic criteria [5], i.e. they were clinically significant strains.

### 2.2. Experimental methods

#### 2.2.1. Mycobacterium culture and preliminary identification

A rapid automatic mycobacterial culture/drug susceptibility testing system (BACTEC MGIT system) or a modified Roche culture method was used for mycobacterial culture in each designated hospital. The preliminary strain identification of MTB and NTM was performed in our hospital using an MPB64 antigen assay and a PNB selective medium.

#### 2.2.2. NTM strain identification

The NTM identified in the initial screening was identified by the Boao gene chip method [6]; if the NTM of a subspecies or species could not be identified, 16S ribosomal ribonucleic acid (*16S rRNA*) [7] and *hsp65* [8] gene sequencing could be used.

#### 2.2.3. NTM susceptibility testing

The broth microdilution method and susceptibility breakpoints recommended by the CLSI were used [4]. Among them, the MAB antimicrobial drug tigecycline had no CLSI recommended breakpoint, so the susceptibility and resistance breakpoints were set to ≤0.5 [9] and ≥8 μg/mL [10], respectively, according to the literature.

##### 2.2.3.1. MAB

The study used RAPMYCO Sensititre^®^ plates. According to the operating instructions, bacteria were ground to adjust the turbidity, and the bacterial solution was automatically inoculated to the plates, which were incubated at 30°*C* ± 2 °C. Depending on the growth of the control wells, the values were read after 3–5 days of incubation. Clarithromycin-susceptible isolates were further incubated on RAPMYCO plates for 14 days to exclude inducible clarithromycin resistance. The plates contained 15 drugs, and the tested concentrations of the drugs were as follows: clarithromycin: 0.06–16 mg/L, amikacin: 1–64 mg/L, linezolid: 1–32 mg/L, moxifloxacin: 0.25–8 mg/L, ciprofloxacin: 0.12–4 mg/L, cefoxitin: 4–128 mg/L, imipenem: 2–64 mg/L, amoxicillin/clavulanic acid: 2/1–64/32 mg/L, tobramycin: 1–16 mg/L, doxycycline: 0.12–16 mg/L, trimethoprim/sulfamethoxazole: 0.25/4.75–8/152 mg/L, tigecycline: 0.015–4 mg/L, minocycline: 1–8 mg/L, cefepime: 1–32 mg/L and ceftriaxone: 4–64 mg/L. The MIC readings showed 80% inhibition of trimethoprim/sulfamethoxazole and 100% inhibition of all other antimicrobial agents. The fast-growing *M. peregrinum* ATCC^®^ 700686 was used as a *Mycobacterium* quality control strain.

##### 2.2.3.2. MAC and *M. kansasii*

SLOMYCO Sensititre^®^ plates were used. According to the operating instructions, bacteria were ground to adjust turbidity, and the bacterial solution was automatically inoculated to the plates, which were incubated at 36°*C* ± 1 °C. Depending on the growth of the control wells, the values were read after 7–14 days of incubation. The plates contained 13 drugs, and the tested concentrations of the drugs were as follows: clarithromycin: 0.06–64 mg/L, amikacin: 1–64 mg/L, linezolid: 1–64 mg/L, moxifloxacin: 0.12–8 mg/L, ciprofloxacin: 0.12–16 mg/L, rifampin: 0.12–8 mg/L, rifabutin: 0.25–8, ethambutol: 0.5–16 mg/L, isoniazid: 0.25–8 mg/L, streptomycin: 0.5–64 mg/L, ethionamide: 0.3–20 mg/L, doxycycline: 0.12–16 mg/L and trimethoprim/sulfamethoxazole: 0.12/2.38–8/152 mg/L. The MIC readings showed 80% inhibition of trimethoprim/sulfamethoxazole and 100% inhibition of all other antimicrobial agents. The slow-growing *M. marinum* ATCC^®^ 927 was used as a *Mycobacterium* quality control strain.

#### 2.2.4. Rules for the interpretation of drug susceptibility plates

##### 2.2.4.1. MAB drug susceptibility reading rules

1. On day 1 of incubation, the presence of bacterial contamination was observed. If growth was observed on day 1, the sensitivity test was repeated. On day 3 of incubation, it was observed whether the positive control holes reached 2+ growth; otherwise, observations were conducted on days 4 and 5.
2. On days 4–5, the MIC values of all drugs except clarithromycin were observed. The value of trimethoprim/sulfamethoxazole was read at 80% pore growth inhibition, while the values of the other drugs were read at the first non-growing pore.
3. On day 14, the clarithromycin results were read, i.e. first pores with complete growth inhibition.
4. If the positive control did not grow well until day 5, the bacterial activity was insufficient, and it was recommended to repeat drug susceptibility testing.

##### 2.2.4.2. MAC and *M. kansasii* drug susceptibility interpretation rules

1. On day 5 of incubation, the presence of miscellaneous bacteria and rapidly growing mycobacteria was observed.
2. On day 7 of incubation, it was observed whether the positive control well had reached 2+ growth; otherwise, observations were conducted on days 10 to 14.
3. If the growth of the positive control well remained poor on day 21, the bacterial activity was insufficient, and it was recommended to repeat drug susceptibility testing.

## 3. Results

### 3.1. Identification of non-tuberculous mycobacterial strains

Among the 81 patients with NTM, a total of 43 clinically common NTM species were identified (excluding duplicate strains, i.e. multiple strains from the same patient), including 43 strains of MAC, 24 strains of MAB and 14 strains of *M. kansasii*.

### 3.2. MAB antimicrobial susceptibility

On days 3–5 of MAB incubation, clarithromycin was susceptible at 87.5%, and the MIC50/MIC90 values were low (0.25/2 μg/mL, respectively); on day 14 of incubation, clarithromycin sensitivity decreased to 50%, and the MIC50/MIC90 values increased significantly (2/>16 μg/mL, respectively). Amikacin had strong antibacterial activity (MIC50/MIC90 were just 2/8 μg/mL, respectively) and was susceptible in 91.6%. Moxifloxacin (95.8%), ciprofloxacin (95.8%), imipenem (95.8%), amoxicillin/clavulanic acid (95.8%), tobramycin (79.1%), doxycycline (95.8%) and trimethoprim/sulfamethoxazole (95.8%) were overwhelmingly drug resistant. Cefoxitin had low antimicrobial activity against MAB (MIC50/MIC90 = 64/128 μg/mL, respectively) and was intermediated (83.3%), drug resistant (16.7%) and non-susceptible; conversely, linezolid was 33.3% susceptible, with high MIC50/MIC90 (16/32 μg/mL) values. There were no MIC breakpoints for tigecycline, minocycline, cefepime and ceftriaxone, among which tigecycline had strong antibacterial activity (MIC50/MIC90 values of just 0.5/2 μg/mL, respectively). When the susceptibility breakpoint was set to ≤0.5 μg/mL according to the literature [9], the sensitivity rate was 50%. When the resistance breakpoint was set to ≥8 μg/mL [10], the resistance rate was 0%. The specific MIC breakpoints and distributions of the MAB antimicrobial drugs are shown in Table 1.

**Table 1.** Susceptibility of *Mycobacterium abscessus* complex to antimicrobial drugs determined.

Susceptibility of *Mycobacterium abscessus* complex to antimicrobial drugs determined by broth microdilution method (*n* = 24)																
Antibacterial drugs	Concentration range of broth microdilution (μg/ml) and the number of strains corresponding to MIC determination *n* (%)											MIC50	MIC90	Susceptible	Intermediate	Resistant
Clarithromycin	MIC	≤0.06	0.12	0.25	0.5	1	2	4	8	16	>16			≤2	4	≥8
3–5 days	*n* (%)	4 (16.7)	5 (20.8)	4 (16.7)	3 (12.5)	4 (16.7)	3 (12.5)	1 (4.2)	0	0	0	0.25	2	21 (87.5)	2 (8.3)	1 (4.2)
14 days	n (%)	2 (8.3)	0	4 (16.7)	4 (16.7)	1 (4.2)	1 (4.2)	2 (8.3)	1 (4.2)	2 (8.3)	7 (29.2)	2	>16	12 (50)	2 (8.3)	10 (41.7)
Amikacin	MIC	≤1	2	4	8	16	32	64	>64			4	8	≤16	32	≥64
	*n* (%)	0	3 (12.5)	12 (50)	7 (29.2)	0	1 (4.2)	0	1 (4.2)					22 (91.6)	1 (4.2)	1 (4.2)
Linezolid	MIC	≤1	2	4	8	16	32	>32				16	32	≤8	16	≥32
	*n* (%)	0	0	5 (20.8)	3 (12.5)	11 (45.8)	5 (20.8)	0						8 (33.3)	11 (45.8)	5 (20.8)
Moxifloxacin	MIC	≤0.25	0.5	1	2	4	8	>8				8	>8	≤1	2	≥4
	*n* (%)	0	0	1 (4.2)	0	4 (16.7)	8 (33.3)	11 (45.8)						1 (4.2)	0	23 (95.8)
Ciprofloxacin	MIC	≤0.12	0.25	0.5	1	2	4	>4				>4	>4	≤1	2	≥4
	*n* (%)	0	0	0	0	1 (4.2)	7 (29.1)	16 (66.7)						0	1 (4.2)	23 (95.8)
Cefoxitin	MIC	≤4	8	16	32	64	128	>128				64	128	≤16	32–64	≥128
	*n* (%)	0	0	0	4 (16.7)	16 (66.7)	2 (8.3)	2 (8.3)						0	20 (83.3)	4 (16.7)
Imipenem	MIC	≤2	4	8	16	32	64	>64				>64	>64	≤4	8–16	≥32
	*n* (%)	0	0	0	1 (4.2)	3 (12.5)	6 (25)	14 (58.3)						0	1 (4.2)	23 (95.8)
Amoxicillin/ Clavulanic acid	MIC	≤2/1	4/2	8/4	16/8	32/16	64/32	>64/32				>64/32	>64/32	≤8/4	16/8	≥32/16
	*n* (%)	0	0	0	1 (4.2)	0	3 (12.5)	20 (83.3)						0	1 (4.2)	23 (95.8)
Tobramycin	MIC	≤1	2	4	8	16	>16					8	>16	≤2	4	≥8
	*n* (%)	1 (4.2)	0	4 (16.7)	14 (58.3)	2 (8.3)	3 (12.5)							1 (4.2)	4 (16.7)	19 (79.1)
Doxycycline	MIC	≤0.12	0.25	0.5	1	2	4	8	16	>16		>16	>16	≤1	2–4	≥8
	*n* (%)	0	0	0	0	0	1 (4.2)	2 (8.3)	2 (8.3)	19 (79.1)				0	1 (4.2)	23 (95.8)
Trimethoprim/ sulfamethoxazole	MIC	≤0.25/4.75	0.5/9.5	1/19	2/38	4/76	8/152	>8/152				>8/152	>8/152	≤2/38	–	≥4/76
	*n* (%)	0	0	0	1 (4.2)	3 (12.5)	7 (29.2)	13 (54.1)						1 (4.2)	0	23 (95.8)
Tigecycline	MIC	≤0.015	0.03	0.06	0.12	0.25	0.5	1	2	4		0.5	2	≤0.5	1–4	≥8
	*n* (%)	0	0	0	0	3 (12.5）	9 (37.5)	7 (29.2)	5 (20.8)	0				12 (50)	12 (50)	0
Minocycline	MIC	≤1	2	4	8	>8						>8	>8	–	–	–
	*n* (%)	0	1 (4.2)	4 (16.7)	0	19 (79.1)										
Cefepime	MIC	≤1	2	4	8	16	32	>32				>32	>32	–	–	–
	*n* (%)	0	0	0	1 (4.2)	0	6 (25)	17 (70.8)								
Ceftriaxone	MIC	≤4	8	16	32	64	>64					>64	>64	–	–	–
	*n* (%)	0	0	1 (4.2)	0	2 (8.3)	21 (87.5)									

### 3.3. MAC antimicrobial susceptibility

The MIC50/MIC90 values of clarithromycin and amikacin were 4/>64 and 16/>64 μg/mL, respectively, with sensitivity rates of 81.4% and 79.1%, respectively. The antibacterial activity of linezolid and moxifloxacin was poor (MIC50/MIC90 = 32/>64 and 4/>8 μg/mL, respectively), and their sensitivity rates were just 20.9% and 9.3%, respectively. Ciprofloxacin, rifampicin, rifabutin, ethambutol, isoniazid, streptomycin, ethionamide, doxycycline and trimethoprim/sulfamethoxazole did not have MIC breakpoints; rifabutin had strong antibacterial activity, with an MIC50 value of only 2 μg/mL, while the rest of the antibacterial drugs had poor antibacterial activity (MIC50/MIC90 were higher). The specific MIC breakpoints and distributions of the MAC antibacterial drugs are shown in Table 2.

**Table 2.** Susceptibility of *Mycobacterium avium* complex to antimicrobial drugs determined.

Susceptibility of *Mycobacterium avium* complex to antimicrobial drugs determined by broth microdilution method (*n* = 43)																		
Antibacterial drugs	Concentration range of broth microdilution (μg/ml) and the number of strains corresponding to MIC determination *n* (%)													MIC50	MIC90	Susceptible	Intermediate	Resistant
Clarithromycin	MIC	≤0.06	0.12	0.25	0.5	1	2	4	8	16	32	64	>64	4	>64	≤8	16	≥32
	*n* (%)	0	1 (2.3)	0	2 (4.7)	0	10 (23.3)	10 (23.3)	12 (27.9)	1 (23)	1 (2.3)	1 (2.3)	5 (11.6)			35 (81.4)	1 (2.3)	7 (16.3)
Amikacin	MIC	≤1	2	4	8	16	32	64	>64					16	>64	≤16	32	≥64
	*n* (%)	0	5 (11.6)	7 (16.3)	5 (11.6)	15 (34.9)	4 (9.3)	3 (6.9)	4 (9.3)							34 (79.1)	2 (4.7)	7 (16.3)
Linezolid	MIC	≤1	2	4	8	16	32	64	>64					32	>64	≤8	16	≥32
	*n* (%)	0	1 (2.3)	0	8 (18.6)	11 (25.6)	12 (27.9)	6 (13.9)	5 (11.6)							9 (20.9)	11 (25.6)	23 (53.5)
Moxifloxacin	MIC	≤0.12	0.25	0.5	1	2	4	8	>8					4	>8	≤1	2	≥4
	*n* (%)	0	0	1 (2.3)	3 (6.9)	9 (20.9)	18 (41.9)	7 (16.3)	5 (11.6)							4 (9.3)	9 (20.9)	30 (69.8)
Ciprofloxacin	MIC	≤0.12	0.25	0.5	1	2	4	8	16	>16				16	>16	–	–	–
	*n* (%)	0	0	0	1 (2.3)	0	3 (6.9)	8 (18.6)	16 (37.2)	15 (34.9)								
Rifampin	MIC	≤0.12	0.25	0.5	1	2	4	8	>8					8	>8	–	–	–
	*n* (%)	0	1 (2.3)	1 (2.3)	7 (16.3)	3 (6.9)	8 (18.6)	10 (23.3)	13 (30.2)									
Rifabutin	MIC	≤0.25	0.5	1	2	4	8	>8						2	>8	–	–	–
	*n* (%)	6 (13.9)	3 (6.9)	13 (30.2)	10 (23.2)	5 (11.6)	1 (2.3)	5 (11.6)										
Ethambutol	MIC	≤0.5	1	2	4	8	16	>16						>16	>16	–	–	–
	*n* (%)	1 (2.3)	0	0	1 (2.3)	11 (25.6)	6 (13.9)	24 (55.8)										
Isoniazid	MIC	≤0.25	0.5	1	2	4	8	>8						>8	>8	–	–	–
	*n* (%)	1 (2.3)	0	0	0	0	4 (9.3)	38 (88.4)										
Streptomycin	MIC	≤0.5	1	2	4	8	16	32	64	>64				16	>64	–	–	–
	*n* (%)	0	2 (4.7)	2 (4.7)	9 (20.9)	3 (6.9)	7 (16.3)	8 (18.6)	5 (11.6)	7 (16.3)								
Ethionamide	MIC	≤0.3	0.6	1.2	2.5	5	10	20	>20					>20	>20	–	–	–
	*n* (%)	0	0	0	0	1 (2.3)	0	1 (2.3)	41 (95.3)									
Doxycycline	MIC	≤0.12	0.25	0.5	1	2	4	8	16	>16				>16	>16	–	–	–
	*n* (%)	0	0	0	0	0	1 (2.3)	0	2 (4.7)	40 (93.0)								
Trimethoprim/ sulfamethoxazole	MIC	≤0.12/2.38	0.25/4.75	0.5/9.5	1/19	2/38	4/76	8/152	>8/152					8/152	>8/152	–	–	–
	*n* (%)	0	0	0	3 (6.9)	4 (9.3)	14 (32.6)	6 (13.9)	16 (37.2)									

### 3.4. *Mycobacterium kansasii* antimicrobial susceptibility

Clarithromycin, amikacin, linezolid, moxifloxacin, rifampicin and rifabutin were all susceptible to *M. kansasii* (100%), with strong antibacterial activity (MIC50/MIC90 were low). Both ciprofloxacin and ethambutol were susceptible at 57.1%, while doxycycline and trimethoprim/sulfamethoxazole were highly resistant (100% and 64.3%, respectively). Although isoniazid, streptomycin and ethionamide did not have MIC breakpoints, they had strong antibacterial activity (MIC50/MIC90 were all low). The specific MIC breakpoints and distribution of the antimicrobial drugs for *M. kansasii* are shown in Table 3.

**Table 3.** Susceptibility of *Mycobacterium kansasii* to antimicrobial drugs determined.

Susceptibility of *Mycobacterium kansasii* to antimicrobial drugs determined by broth microdilution method (*n* = 14)																	
Antibacterial drugs	Concentration range of broth microdilution (μg/ml) and the number of strains corresponding to MIC determination *n* (%)												MIC50	MIC90	Susceptible	Intermediate	Resistant
Clarithromycin	MIC	≤0.06	0.12	0.25	0.5	1	2	4	8	16	32	64	0.5	0.5	≤8	16	≥32
	*n* (%)	0	1 (7.1)	2 (14.9)	11 (78.6)	0	0	0	0	0	0	0			14 (100)	0	0
Amikacin	MIC	≤1	2	4	8	16	32	64					4	4	≤16	32	≥64
	*n* (%)	1 (7.1)	4 (28.6)	8 (57.1)	1 (7.1)	0	0	0							14 (100)	0	0
Linezolid	MIC	≤1	2	4	8	16	32	64					2	2	≤8	16	≥32
	*n* (%)	5 (35.7)	9 (64.3)	0	0	0	0	0							14 (100)	0	0
Moxifloxacin	MIC	≤0.12	0.25	0.5	1	2	4	8					≤0.12	0.25	≤1	2	≥4
	*n* (%)	12 (85.7)	2 (14.3)	0	0		0	0							14 (100)	0	0
Ciprofloxacin	MIC	≤0.12	0.25	0.5	1	2	4	8	16				1	2	≤1	2	≥4
	*n* (%)	0	0	3 (21.4)	5 (35.7)	6 (42.9)	0	0	0						8 (57.1)	6 (42.9)	0
Rifampin	MIC	≤0.12	0.25	0.5	1	2	4	8					≤0.12	0.25	≤1	–	≥2
	*n* (%)	10 (71.4)	3 (21.4)	1 (7.1)	0	0	0	0							14 (100)	0	0
Rifabutin	MIC	≤0.25	0.5	1	2	4	8						≤0.25	≤0.25	≤2	–	≥4
	*n* (%)	14 (100)	0	0	0	0	0								14 (100)	0	0
Ethambutol	MIC	≤0.5	1	2	4	8	16						2	16	≤4	–	>4
	*n* (%)	0	1 (7.1)	7 (50)	0	1 (7.1)	5 (35.7)								8 (57.1)		6 (42.9)
Doxycycline	MIC	≤0.12	0.25	0.5	1	2	4	8	16	>16			>16	>16	≤1	2–4	≥8
	*n* (%)	0	0	0	0	0	0	0	1 (7.1)	13 (92.9)					0	0	14 (100)
Trimethoprim/ sulfamethoxazole	MIC	≤0.12/2.38	0.25/4.75	0.5/9.5	1/19	2/38	4/76	8/152	>8/152				4/76	>8/152	≤2/38	–	≥4/76
	*n* (%)	4 (28.6)	0	0	0	1 (7.1)	2 (14.3)	2 (14.3)	5 (35.7)						5 (35.7)	0	9 (64.3)
Isoniazid	MIC	≤0.25	0.5	1	2	4	8						1	1			
	*n* (%)	0	4 (28.6)	9 (64.3)	1 (7.1)	0	0										
Streptomycin	MIC	≤0.5	1	2	4	8	16	32	64				4	8			
	*n* (%)	0	0	4 (40)	7 (40)	3 (20)	0	0	0								
Ethionamide	MIC	≤0.3	0.6	1.2	2.5	5	10	20					≤0.3	≤0.3			
	*n* (%)	13 (92.9)	0	1 (7.1)	0	0	0	0									

## 4. Discussion

The most common species causing NTM lung disease are, in order, MAC, MAB and *M. kansasii.* In particular, MAC and MAB lung diseases have low overall treatment success rates, and different studies have found that they are highly resistant to most antibacterial drugs. In the present study, MAC was found to be highly susceptible to clarithromycin and amikacin, while MAB had high susceptibility to amikacin only.

Of all NTM lung diseases, MAB is the most difficult to treat because of the species’ high level of drug resistance, which makes it difficult to develop effective regimens. Recent NTM international treatment guidelines for pulmonary disease [1] address the selection of drug therapy for *M. abscessus* pulmonary disease and recommend that regimens be developed first based on the results of macrolide drug sensitivity tests. The MAB complex is divided into *abscessus*, *bolletii* and *massiliense* subspecies, of which *abscessus* and *bolletii* have two mechanisms leading to macrolide resistance [11,12]. The first two subspecies have functional *erm(41)* genes, while the *massiliense* subspecies has no functional *erm(41)* genes and remain macrolide susceptible at the first macrolide treatment [13]. Induced resistance to macrolides in MAB was reported to be 68.4% and 74.3% for the *abscessus* and *bolletii* subspecies, respectively, while no induced resistance was found in the *massiliense* subspecies [14].

In this study, we found that MAB had high sensitivity to clarithromycin (87.5%) for 3–5 days of incubation, while 41.7% developed induced resistance up to 14 days of incubation, which was higher than the figure reported by domestic authors (39.48%) [15]. Some *abscessus* and *bolletii* subspecies isolates were reported to have *erm*(41) T28C mutation, resulting in a non-functional gene that instead remains macrolide sensitive [16]. It has also been reported [17] that some *massiliense* subspecies isolates have functional *erm(41)* genes, resulting in induced resistance to macrolides. Therefore, the identification of *abscessus* subspecies may not be a true predictor of the occurrence of macrolide resistance, suggesting that a phenotypic drug sensitivity test or *erm* sequencing is essential for predicting resistance. Induced resistance or acquired mutational resistance to macrolides can lead to a significant reduction in the treatment success for MAB lung disease [18–20]. In the present study, amikacin had a low drug resistance rate (4.2%) and strong antibacterial activity against MAB (MIC50/MIC90 was only 4/8 μg/mL, respectively). Recently, domestic scholars [14,15] reported that MAB also had low drug resistance rates against amikacin (3.9% and 3.51%); however, the MIC50/MIC90 (16/32 and 8/16 μg/mL, respectively) values were significantly higher than in our study, which may be related to the epidemic strains or drug exposure in different regions. We found that in addition to amikacin having a low drug resistance rate and strong antibacterial activity against MAB, cefoxitin and linezolid – which are among the drugs recommended in international guidelines – had low rates of drug resistance (16.7% and 20.8%, respectively); however, most strains were intermediate (83.3% and 45.8%, respectively), while imipenem was almost resistant (95.8%). The MIC50/MIC90 values of cefoxitin, linezolid and imipenem were higher (64/128, 16/32 and >64/>64 µg/mL, respectively), indicating weak antibacterial activity, which was basically consistent with the values in domestic and foreign reports [14,15,21]. Especially for macrolide-resistant MAB lung disease, it is difficult to combine effective regimens.

Although there was no CLSI-recommended resistance breakpoint for tigecycline, a highly sensitive breakpoint was set according to the literature. The key was strong antibacterial activity (MIC50/MIC90 = 0.5/2 μg/mL), which was consistent with recent reports [14,15,21], suggesting that it can be included in regimens as an effective drug. However, *in vitro* MIC and *in vivo* efficacy need to be confirmed by further clinical studies. Moxifloxacin was not recommended in the guidelines, but it is widely used in clinical practice. Our study found that moxifloxacin had a high drug resistance rate (95.8%), with MIC50/MIC90 = 8/>8 μg/mL, suggesting weak antibacterial activity, which was consistent with several studies [14,15,21]. Other scholars [22] determined the susceptibility breakpoint of moxifloxacin to be 0.25 μg/mL *via* PK/PD studies; the effective concentration could not be reached even when the dose was increased to 800 mg qd, suggesting that the efficacy of the conventional dose was limited.

Overall, it is difficult to combine effective regimens among the currently available guideline-recommended drugs, and new drug development and clinical studies of synergistic drug combination regimens are required. Facilitating the rapid identification of new drugs can be achieved by the thorough screening of various large chemical libraries from pharmaceutical and other scientific laboratories around the world [23].

*Mycobacterium avium* complex lung disease is second only to *M. abscessus* lung disease in terms of difficulty of treatment, especially for cavitary and refractory MAC lung disease [24]. International guidelines [1] recommended drug therapy options for MAC lung disease, and although the CLSI gives sensitivity breakpoints for linezolid and moxifloxacin, the correlation between *in vivo* and *in vitro* efficacy has not been established [25]. The combination of moxifloxacin in a macrolide-containing regimen has not been shown to increase efficacy [26] and may instead increase the emergence of macrolide-resistant mutations [27]. It is known that macrolides are the core drugs in the treatment of MAC lung disease and that treatment success is greatly reduced by the development of resistance [28,29].

No susceptibility breakpoint was given for rifampicin and ethambutol in the standard triple combination of antimicrobial drugs recommended by the guidelines. One study [30] reported that MIC ≥ 8 μg/mL for rifampicin and ethambutol was negatively correlated with *in vivo* efficacy, suggesting that it could be used as a breakpoint for resistance. However, further clinical studies are needed to confirm this.

A systematic review [31] found that the ATS-recommended standard triple regimen for patients with macrolide-susceptible MAC lung disease was superior to other macrolide-containing regimens. Another study [29] found that ethambutol rejection was an important risk factor for acquired drug resistance and the treatment failure of macrolides, suggesting that it may be related to the synergistic effect of rifampicin and ethambutol combined with macrolides. In this study, the MIC50 value (2 μg/mL) for rifabutin was significantly lower than for rifampicin, suggesting strong antibacterial activity. When MIC ≥ 8 µg/mL was used as the drug resistance breakpoint, the drug resistance rate was only 13.9%, and the interaction between rifabutin and macrolides had less influence than that of rifampicin [32]; accordingly, rifabutin could be used as a preference among rifamycins; however, the correlation between *in vitro* MIC and *in vivo* efficacy is unclear, and rifabutin’s superiority to rifampicin requires confirmation in further clinical studies.

Overall, MAC is resistant to most antimicrobial drugs to varying degrees, and it is difficult to combine effective regimens if macrolide resistance is present.

*Mycobacterium kansasii* lung disease has a higher success rate of treatment and a relatively lower degree of drug resistance than the first two lung diseases. International guidelines [1] have recommended triple-combination drugs, such as azithromycin (or clarithromycin), rifampicin (or rifabutin), ethambutol or isoniazid as the preferred choice for *M. kansasii* pneumonia. The antibacterial activity of the drugs in the standard triple regimen was found to be strong in this study, implying that the success rate of treatment for *M. kansasii* lung disease may be high. The drug resistance rate of ethambutol was 42.9%, and the MIC50/MIC90 was 2/16 μg/mL, suggesting poor antibacterial activity. However, ethambutol was preferred as an accompanying drug in the standard rifampicin-containing regimen in international guidelines [1]. The third edition of the CLSI drug sensitivity guidelines [33] removed the sensitivity breakpoint and gave only MIC for clinical reference; considering the poor reproducibility of ethambutol drug sensitivity and the uncertainty of the correlation between *in vitro* MIC and *in vivo* efficacy, this may be misleading to the clinic. Overall, *M. kansasii* pneumonia is susceptible to most antimicrobial drugs, with strong antimicrobial activity and a high cure rate.

This study has some limitations. First, the sample size of NTM drug sensitivity in this study was small; accordingly, the next step would be to expand the sample size to further investigate drug sensitivity in this region. Second, MAB was not further identified at the subspecies level; the next step would be to identify it at the subspecies level to further analyse the drug sensitivity of different subspecies. At the same time, the combination of *16S rRNA* gene sequencing and *rpoB* markers was considered to identify NTM to improve identification ability [34]. Although this study has some limitations, it can provide a reference basis for the clinical selection of drugs in this region.

## 5. Conclusion

In conclusion, the MIC distribution and degree of resistance of three common respiratory NTM species differed significantly. Most antimicrobial drugs were significantly less resistant to *M. kansasii* than MAB and MAC and had stronger antibacterial activity. The reference for *in vitro* drug susceptibility is extremely important for developing an effective starting regimen for treating MAB and MAC lung disease. Given the current high degree of antimicrobial drug resistance, there is an urgent need for the development of new drugs and clinical trials of new synergistic drug combination regimens to improve clinical efficacy.

## Data Availability

All data generated or analysed during this study are included in this published article.
